# Prevalence, Laterality, and Classification of Ossified Petroclival Ligaments: An Anatomical and Histological Study With Application to Skull Base Surgery

**DOI:** 10.7759/cureus.36469

**Published:** 2023-03-21

**Authors:** Uduak-Obong I Ekanem, Arada Chaiyamoon, Juan J Cardona, J. Franklin Berry, Grzegorz Wysiadecki, Jerzy A Walocha, Joe Iwanaga, Aaron S Dumont, R. Shane Tubbs

**Affiliations:** 1 Department of Anatomy, Tulane University School of Medicine, New Orleans, USA; 2 Department of Anatomy, Faculty of Medicine, Khon Kaen University, Khon Kaen, THA; 3 Department of Neurosurgery, Tulane University School of Medicine, New Orleans, USA; 4 Department of Anatomy and Histology, Medical University of Poland, Lodz, POL; 5 Department of Anatomy, Jagiellonian University Medical College, Krakow, POL; 6 Department of Anatomical Sciences, St. George’s University, St. George’s, GRD; 7 Department of Neurosurgery and Structural & Cellular Biology, Tulane University School of Medicine, New Orleans, USA; 8 Neurosurgery and Ochsner Neuroscience Institute, Ochsner Health System, New Orleans, USA

**Keywords:** ossification, skull base, petrosphenoidal ligament, dorello’s canal, cranium, imaging, neurosurgery, anatomic variation

## Abstract

Background

The petroclival ligament (PL) forms the roof of Dorello’s canal (DC). In humans, partial and complete ossification of this ligament have been reported. When completely ossified, DC is transformed into a bony foramen for the abducens nerve and accompanying vascular structures. As this osteological finding might have an impact on skull base surgery, this anatomical study was performed.

Methodology

Using 100 adult human skulls, the presence of an ossified PL was noted and classified. The diameter of the resultant bony foramen and laterality were documented. Additionally, PL was evaluated histologically in 10 heads.

Results

Overall, 8% of the sides were found to have partial or complete ossification of the PL. Partial ossification (type I) was noted on 3% of the sides. Completely ossified PL was identified on 5% of the sides. Some ossified ligaments (2.5%) were seen as an ossified bridge (type II), and others (2.5%) were converted into small foramina (type III). Three skulls (3%) were found to have a completely ossified ligament bilaterally. The mean diameter of the underlying DC was 0.8 mm. Partially ossified ligaments were statistically more likely to be on the right sides, and the diameter of the underlying DC was statistically smaller in type III. Histologically, the PL was found to have bone within it on three skull sides.

Conclusions

An ossified ligament can be found on imaging of the skull base. Moreover, during surgical approaches to the petroclival region and, specifically, DC, skull base surgeons should be cognizant of this anatomical variation.

## Introduction

The petroclival ligament (also called the petrosphenoidal, petroclinoid, superior sphenopetrosal, and Gruber’s ligament) extends from the petrous apex to the lateral edge of the clivus. This ligament forms the superolateral border of Dorello’s canal and lies within the inferomedial paraclival triangle [1-3]. Typically, the ligament creates the roof of Dorello’s canal, which is a passageway for the abducens nerve (CN VI), the dorsal meningeal artery, and the inferior petrosal sinus [1-10]. This ligament can vary in both size and type, with lengths reported in the range of 4-14 mm, and may be characterized as hypoplastic, fragmented, or ossified [1,3,4,6-9]. The petroclival ligament is implicated in many clinical processes owing to its location, but, most importantly, due to its potential to affect the abducens nerve. Abducens nerve paresis, pituitary adenomas, skull base metastases, meningiomas, petrous apicitis, herniation syndromes, cerebral aneurysms, and even head trauma have been reported to involve the petroclival ligament [2,6,7,9,11]. Given that the petroclival ligament serves as an important landmark in locating the abducens nerve and other structures during skull base surgery, it is no surprise that the ligament may play a key role in several different pathological neurological processes. There have been multiple anatomical studies of the petroclival ligament. Our goal was to expand on current knowledge, focusing on the prevalence and laterality of its possible ossification. Ossification allows or enhances attachment of the abducens nerve within Dorello’s canal [9]. Ossified petroclival ligaments (variably referred to as the foramen petrosphenoideum anomalum, sphenopetrous bridge, or bony abducens bridge [4]) have been linked to neural impingement syndromes, among other pathologies. This and other considerations underscore the importance of this anatomical study.

## Materials and methods

**Figure 1:**
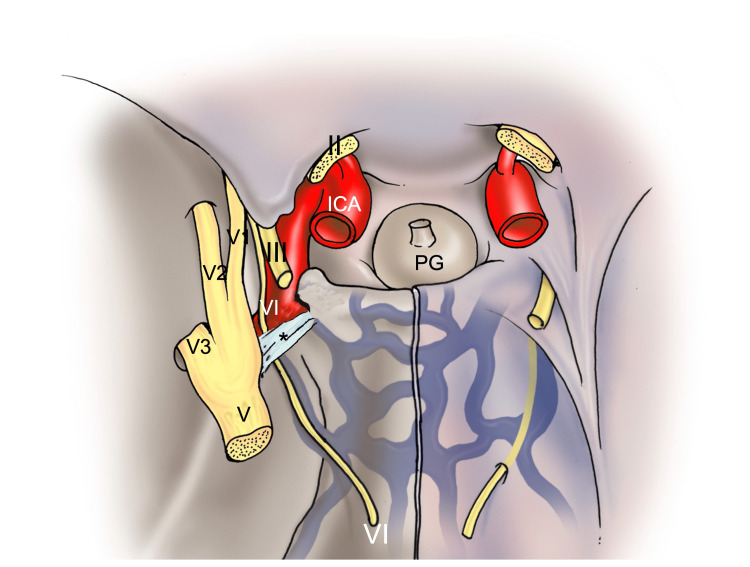
Schematic drawing of the petroclival ligament (*). Note the abducens nerve (VI), oculomotor nerve (III), optic nerve (II), trigeminal nerve (V), and internal carotid artery (ICA).

**Figure 2:**
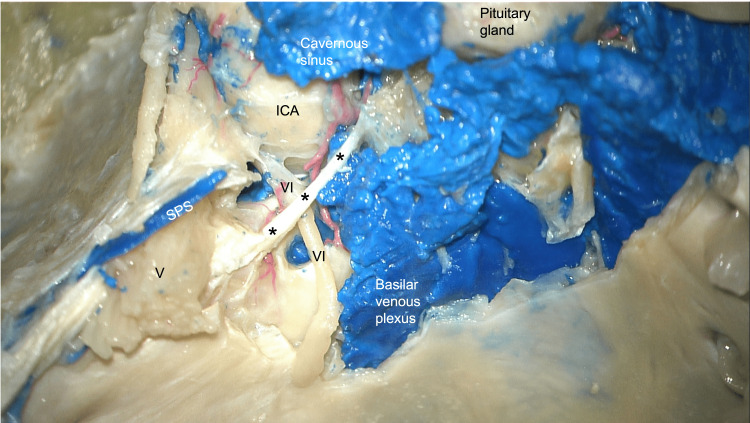
Dissection of the skull base in a cadaveric specimen. The petroclival ligament is seen at the *. Note the abducens nerve (VI), trigeminal nerve (V), superior petrosal sinus (SPS), and internal carotid artery (ICA).

Using 100 adult human skulls (200 sides), the presence of an ossified petroclival ligament was noted. The prevalence among these specimens and laterality were documented. The sex and exact ages of the skulls were not known. However, the estimated age range for the cohort was determined to be between 30 and 80 years old. The skulls were derived from a North American sample. In each skull found to have an ossified petroclival ligament, the diameter of the underlying Dorello’s canal was measured using digital calipers (Mitutoyo, Japan). All dissections were performed using a surgical microscope (Zeiss, Germany). A classification system was developed to better describe the anatomical variations found among these ossified ligaments and their influence on the size of Dorello’s canal. The classification system used was as follows: partially ossified ligament = type I; ossified bridge = type II; and Dorello’s canal converted to a small foramen = type III. Laterality of the ossified petroclival ligaments was analyzed using the chi-square test, and measurements between sides were analyzed with Student’s t-test with significance set at p-values <0.05 (Wizard). Additionally, in 10 cadaveric human heads (six males and four females) with a mean age at death of 69 years (58-87 years), the petroclival ligament was harvested and evaluated histologically with hematoxylin and eosin and Masson trichrome staining (Figures 1, 2). If necessary, specimens were decalcified using hydrochloric acid (Epredia, Portsmouth, NH, USA).

## Results

**Figure 3:**
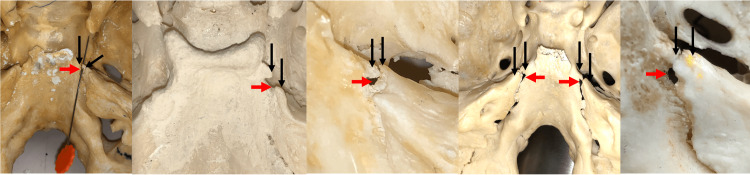
Skull examples of the type I ossified petroclival ligaments. As partially ossified bony bridges, the two edges of the bony growth of the ligament are seen at the black arrows. Dorello’s canal is shown at the red arrows. Note the fourth image has bilateral bridging.

**Figure 4:**
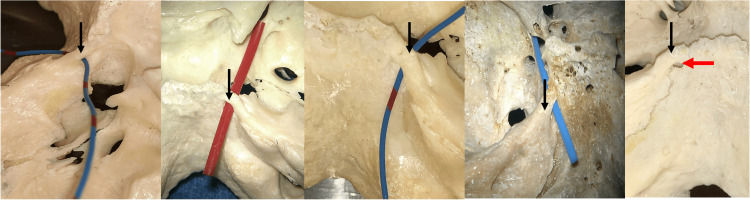
Skull examples of type II ossified petroclival ligaments (black arrows). Dorello’s canal is seen at the red arrow or is occupied by vessel loops.

**Figure 5:**
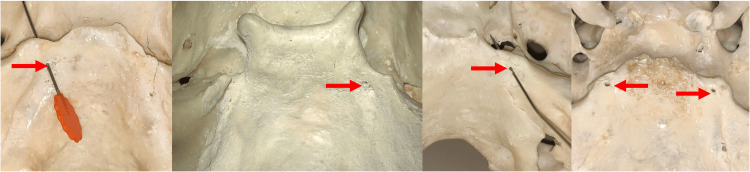
Skull examples of type III ossified petroclival ligaments. Note the resultant small Dorello’s canal at the red arrows and a bilateral occurrence on the fourth image.

**Figure 6:**
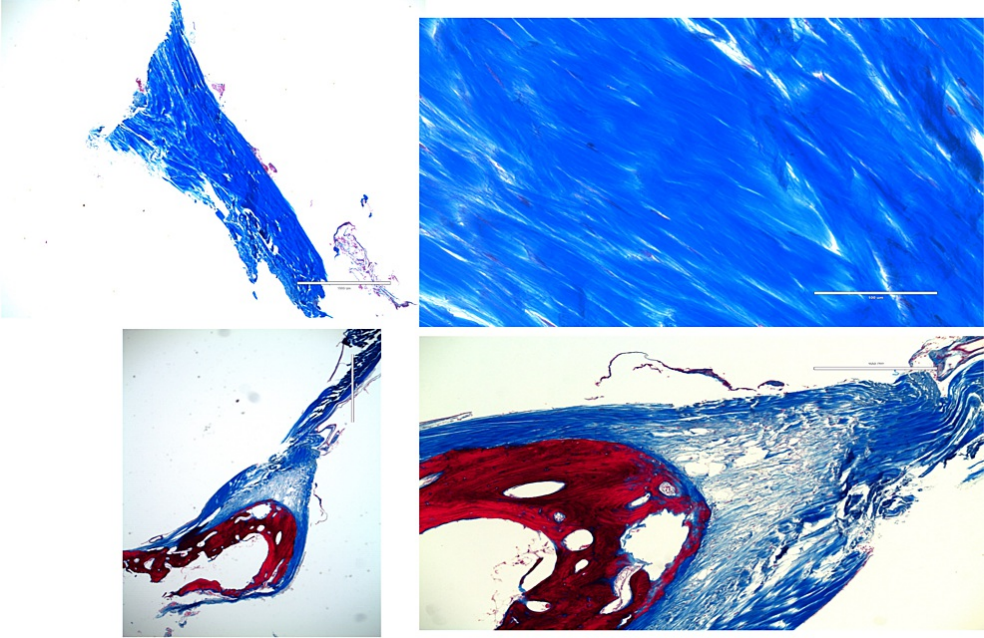
Histological examples (Masson trichrome) of normal petroclival ligaments (upper images) and partially ossified ligaments (lower images). Here, the connective tissue is stained blue and the bone is stained red.

**Figure 7:**
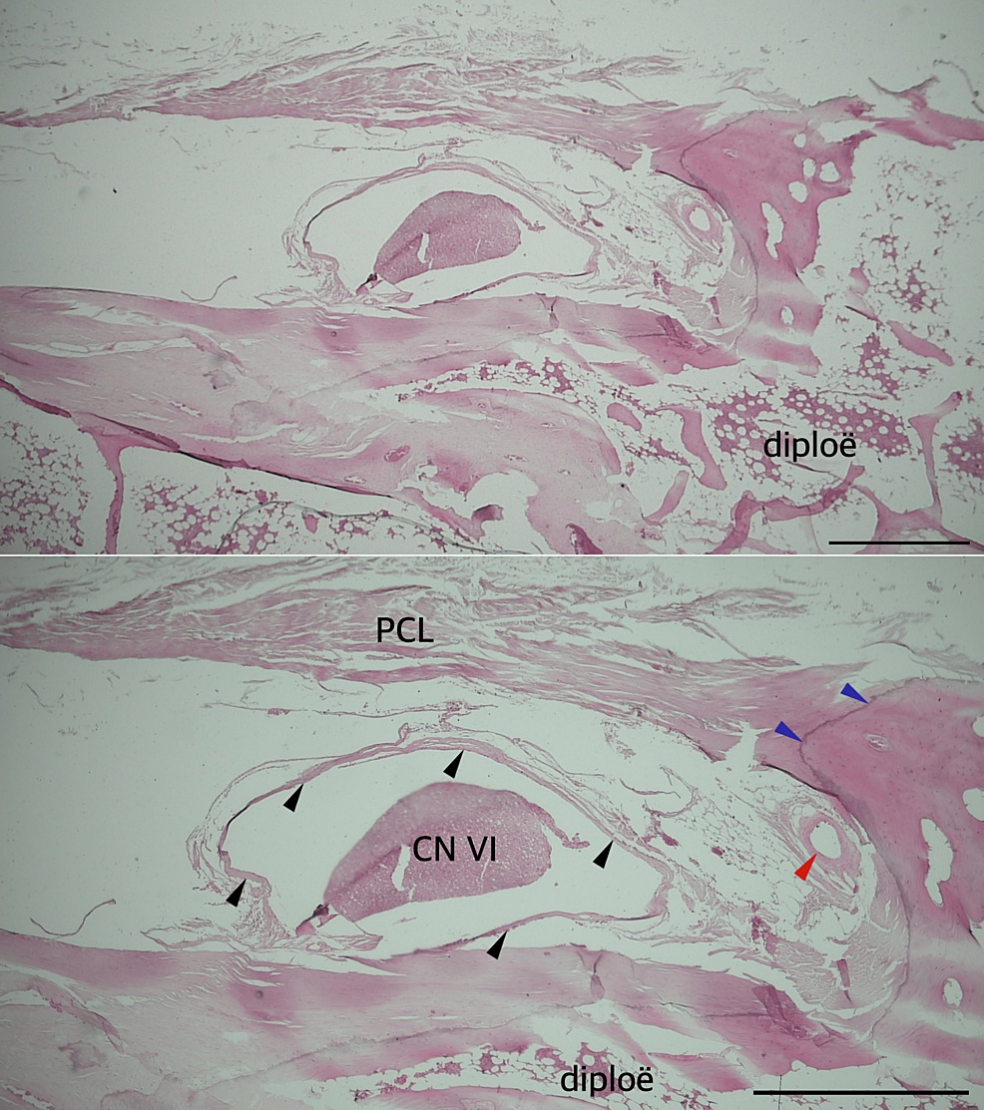
Histological specimen showing Dorello’s canal and petroclival ligament (PCL) with partial ossification. Hematoxylin and eosin stain. Black arrowheads show the dural sleeve of the abducens nerve (CN VI). Blue arrowheads indicate partial ossification of the PCL. A red arrowhead marks a branch of the dorsal meningeal artery.

A total of 16 (8%) sides were found to have partial or complete ossification of the petroclival ligament. Partial ossification (type I) was noted on 3% of sides (6/200; five right sides and one left side) (Figure 3). One skull (1%) was found to have partial ossification bilaterally. In the partially ossified ligaments, components arising from the petrous part of the temporal bone and components arising from the clivus contributed more or less equally to the ossification. A completely ossified petroclival ligament was identified on 5% of sides (10/200; five left and five right sides). Of the completely ossified ligaments, some (2.5%) were seen as an ossified bridge (5/200 sides) (type II) (Figure 4), and others (2.5%) were converted to small foramina (5/200 sides) (type III) (Figure 5). Three skulls (3%) were found to have a completely ossified ligament bilaterally. The mean diameter of the underlying Dorello’s canal was 0.8 mm with a range of 0.3 to 2.2 mm. Partially ossified ligaments were statistically more likely to be on the right sides, and the diameter of the underlying Dorello’s canal was statistically (p < 0.05) smaller in type III ossified petroclival ligaments. Statistically, partial ossifications were more likely to be on the right versus left sides. In specimens that underwent histological analysis (Figures 6, 7), the ligament was found to have bone within it on three skull sides (two left and one right). In all three of these ligaments, the ossification was in the posterior aspect of the ligament as it attached to the petrous part of the temporal bone (Figure 6).

## Discussion

Rigid “anatomical norms” may falsely create an idealized image of the human body which might not always be true in reality; hence, the need for reporting anatomical variations [12]. This is especially true of the anatomical variations of the intracranial ligaments. In this study, we examined the ossification of the petroclival ligament. The clinical and surgical ramifications of such an ossified ligament are obvious. Interestingly, in most non-human primates, Dorello’s canal is normally completely bony, i.e., the petroclival ligament is ossified and the canal exists as a foramen [13].

Clinical aspects

An abducens nerve palsy may be caused by several different mechanisms. A possible common factor is its greater angulation as it passes through Dorello’s canal [10]. In the canal, greater angulation of the abducens nerve is due to its tight fixation via dense adhesions between its dural sheath and the endosteal dura of the petrous apex and due to possible ossification of the petroclival ligament. Mechanisms such as intracranial hypertension, intracranial hypotension, and cerebrospinal fluid flow are major contributors to the stretching of the abducens nerve on this fixation point after downward displacement of the brainstem [1,6,7,9,10,14]. Ossification of the petroclival ligament and this laterality can be explored as another factor contributing to abducens nerve paralysis; increased ossification can put additional stress on the abducens nerve through narrowing of Dorello’s canal, increased lateralization of the nerve, and compression by surrounding structures running with the nerve, such as the dorsal meningeal artery [9,13].

Prevalence

In skull specimens, we identified either partially or completely ossified petroclival ligament in 8% of 200 skull sides. In a Greek population, Tsitsopoulos et al. reported ossification of the petroclival ligament in 10% of 20 cadavers and on 31 cadaver sides, whereas Iwanaga et al. found 5.6% with ossified ligaments [15,16]. Iaconetta et al. found it ossified in three German specimens but did not report the total number studied [17]. From North America, Barges-Coll et al. reported the ligament ossified in 10% of specimens, and Destrieux et al., in a French study, found it ossified in 3.6% of specimens [18,19]. In an Israeli study, Umansky et al. found ossification of the ligament in 10% of cadavers [20]. Regarding the radiographic identification of ossified ligaments, Özgür et al., from Turkey, identified 46 patients (32 men, 14 women) with ossification of the petroclival ligament from 523 head CTs (8.8%) [13]. In addition, using CT, Inal et al., from Turkey, found for the right petroclival ligament, there were partial ossifications in 9.8% and complete ossification in 2.3% [21]. Partial ossification was found in 9.8% and partial ossification in 2.9%. In a study from the United Kingdom, Touska et al. found ossification of the ligament on 10.8% of CT images [22]. However, the ossified ligament might not be seen on imaging due to its small size and can depend on the thickness of the imaging slices. Moreover, some have stated that ossifications in this region, e.g., the petroclival ligament cannot be distinguished on CT, therefore bringing into question the findings of imaging studies of this structure [21]. Therefore, our finding of 8% is in line with other reports in the literature of partial or complete ossification of the petroclival ligament ranging from 2.3% to 10.8% [13,15,17-22]. Lastly, as some ossification might be found in these ligaments histologically, as shown in this study, many of the previous reports have probably underestimated the true prevalence of ligament ossification.

Laterality

In a study by Özgür et al., 6.5% of petroclival ligament ossifications were on the right and 5.1% were on the left, and of these, 3.6% were partial and 2.2% were complete. In skulls, Peker et al., in a Turkish cohort, found partially ossified ligaments on 7.6% of the right sides and 10.1% of the left sides [23]. We found partial ossification (type I) ligaments on 3% of sides with five of these on the right side and one on the left side. A completely ossified petroclival ligament was identified on 5% of sides, with five of these on the left side and five on the right side. Therefore, statistically, it was more likely for a partial ossification to be found on the right side.

Surgery

The petroclival ligament is a key landmark in skull base approaches of endonasal, transclival, transsellar, transcavernous, or paramedian types, as well as endovascular procedures. Damage to the abducens nerve during skull base surgery can occur due to the fixation of the nerve to the overlying petroclival ligament and may result in abducens nerve palsy. Petroclival tumors have been reported to displace the abducens nerve, and stretching of the nerve can be compounded in cases of partial or complete ossification of the petroclival ligament [8]. With this knowledge, the compound effect of lateral displacement of the nerve in contributing to abducens nerve paralysis should be considered in surgical applications. Therefore, the classification system used in this study can be a potential tool for helping to ensure the protection of the abducens nerve based on preoperative imaging or intraoperative findings, e.g., a type III ossified ligament which results in a smaller Dorello’s canal.

Acknowledgments and ethics statement

The authors sincerely thank those who donated their bodies to science so that anatomical research could be performed. Results from such research can potentially increase mankind’s overall knowledge which can then improve patient care. Therefore, these donors and their families deserve our highest gratitude [24]. The authors state that every effort was made to follow all local and international ethical guidelines and laws that pertain to the use of human cadaveric donors in anatomical research [25].

## Conclusions

Reports in the literature show a variable range for the prevalence of an ossified petroclival ligament. The possibility of ossification of the petroclival ligament has important ramifications for the contents of Dorello’s canal. As such, during surgical approaches to the petroclival region and, specifically, Dorello’s canal, the skull base surgeon should be cognizant of this important anatomical variation and its possible consequences.
